# Tilianin Protects against Ischemia/Reperfusion-Induced Myocardial Injury through the Inhibition of the Ca^2+^/Calmodulin-Dependent Protein Kinase II-Dependent Apoptotic and Inflammatory Signaling Pathways

**DOI:** 10.1155/2020/5939715

**Published:** 2020-10-09

**Authors:** Hailun Jiang, Jianguo Xing, Jiansong Fang, Linlin Wang, Yu Wang, Li Zeng, Zhuorong Li, Rui Liu

**Affiliations:** ^1^Institute of Medicinal Biotechnology, Chinese Academy of Medical Sciences and Peking Union Medical College, Beijing 100050, China; ^2^Key Laboratory of Uighur Medicine of Xinjiang Uygur Autonomous Region, Xinjiang Institute of Materia Medica, Urumqi, 830004, China; ^3^Institute of Clinical Pharmacology, Guangzhou University of Chinese Medicine, Guangzhou 510405, China; ^4^College of Medical, Veterinary and Life Sciences, University of Glasgow, Glasgow G12 8QQ, UK

## Abstract

Tilianin is a naturally occurring phenolic compound with a cardioprotective effect against myocardial ischemia/reperfusion injury (MIRI). The aim of our study was to determine the potential targets and mechanism of action of tilianin against cardiac injury induced by MIRI. An *in silico* docking model was used in this study for binding mode analysis between tilianin and Ca^2+^/calmodulin-dependent protein kinase II (CaMKII). Oxygen-glucose deprivation/reperfusion- (OGD/R-) injured H9c2 cardiomyocytes and ischemia/reperfusion- (I/R-) injured isolated rat hearts were developed as *in vitro* and *ex vivo* models, respectively, which were both treated with tilianin in the absence or presence of a specific CaMKII inhibitor KN93 for target verification and mechanistic exploration. Results demonstrated the ability of tilianin to facilitater the recovery of OGD/R-induced cardiomyocyte injury and the maintenance of cardiac function in I/R-injured hearts. Tilianin interacted with CaMKII*δ* with an efficient binding performance, a favorable binding score, and restraining p-CaMKII and ox-CaMKII expression in cardiomyocytes injured by MIRI. Importantly, inhibition of CaMKII abolished tilianin-mediated recovery of OGD/R-induced cardiomyocyte injury and maintenance of cardiac function in I/R-injured hearts, accompanied by the disability to protect mitochondrial function. Furthermore, the protective effects of tilianin towards mitochondrion-associated proapoptotic and antiapoptotic protein counterbalance and c-Jun N-terminal kinase (JNK)/nuclear factor- (NF-) *κ*B-related inflammation suppression were both abolished after pharmacological inhibition of CaMKII. Our investigation indicated that the inhibition of CaMKII-mediated mitochondrial apoptosis and JNK/NF-*κ*B inflammation might be considered as a pivotal mechanism used by tilianin to exert its protective effects on MIRI cardiac damage.

## 1. Introduction

Cardiac reperfusion following acute myocardial ischemia, known as myocardial ischemia/reperfusion injury (MIRI), contributes to cardiomyocyte damage. Cardiac myocyte injury in response to ischemia/reperfusion (I/R) is a complex process involving many mechanisms. Among the intracellular signaling pathways that are stimulated in MIRI, Ca^2+^/calmodulin-dependent protein kinase II (CaMKII) has been documented as being a critical mediator of this maladaptive response [1]. CaMKII*δ*, one of the four *α*, *β*, *γ*, and *δ* isoforms, is predominantly expressed in the heart with the splice variant *δ*C residing in the cytosol. CaMKII is specifically located in the mitochondria of cardiomyocytes. During postischemic reperfusion, both cellular Ca^2+^ and ROS activate CaMKII by binding with diverse activation sites and to a greater extent in the initial period of reperfusion [2–5]. The phosphorylation and oxidation of CaMKII induce mitochondrial damage and regulate critical proteins related to the apoptotic and inflammatory progress due to MIRI [6–10]. Therefore, CaMKII may represent an attractive target for reducing mitochondrial dysfunction and inflammatory response induced by MIRI.

Tilianin is a flavonoid glycoside present in two medicinal and edible plants, *Dracocephalum moldavica* and *Agastache rugosa*, which are widely used as traditional Chinese medicines. In addition, they are nutritional foods regularly consumed as healthy drinks combined with sugar or honey. Tilianin possesses significant anti-inflammatory and cardiovascular protective properties including energy metabolism regulation, anti-hypoxic, and anti-atherogenesis effects [6, 11]. More importantly, tilianin exerts cardioprotective effects in experimental models of MIRI [11–14], associated with the improvement of mitochondrial function [11, 15], suppression of the myocardial apoptotic pathway, and reduction of the inflammatory response [14]. We obtained the patent for the extraction and purification of tilianin from *Dracocephalum moldavica* L. and received the support of a medical staff to evaluate its effect as a MIRI therapy approved by the Major Scientific and Technological Special Project for “Significant New Drugs Creation” of China. However, the tilianin mechanism of action against MIRI-induced cardiac damage is still not clear.

In our recent report, CaMKII*α* isoform has been shown to be a potential target of tilianin to exert protection against neuronal damage due to oxygen-glucose deprivation/reperfusion (OGD/R) injury *in vitro* [16]. Despite the cardioprotective activity of tilianin, scarce evidence is available regarding the correlation between tilianin and CaMKII*δ*, the heart-specific isoform, in the pathology of MIRI-induced cardiac injury. Thus, in this study, the role of CaMKII*δ* in tilianin-mediated cardioprotective effect due to MIRI was investigated, and the underlying molecular mechanism used by tilianin in the CaMKII signaling pathway was explored both *in vitro* using OGD/R-injured H9c2 cardiomyocytes and *ex vivo* using an I/R-induced isolated Sprague-Dawley (SD) rat heart through Langendorff perfusion.

## 2. Materials and Methods

### 2.1. Cell Culture and Treatments

H9c2 cardiomyocytes, derived from the embryonic BD1X rat heart tissue, were purchased from the American Type Culture Collection (ATCC, Manassas, VA, USA). Cells were cultured in high-glucose Dulbecco's modified Eagle medium (DMEM) (Invitrogen, Carlsbad, CA, USA) supplemented with 10% fetal bovine serum (FBS) (Invitrogen) and 2 mM GlutaMAX (Invitrogen) and incubated at 37°C in a humidified atmosphere containing 5% CO_2_.

The OGD/R procedure was performed according to previous studies with a slight modification [17, 18]. Briefly, H9c2 cardiomyocytes were cultured in glucose-free DMEM containing 5 mM sodium dithionite (Na_2_S_2_O_4_) for 2 h to induce OGD. The supernatant was then replaced with complete medium and incubated for 6 h to mimic reoxygenation.

**Figure 1:**
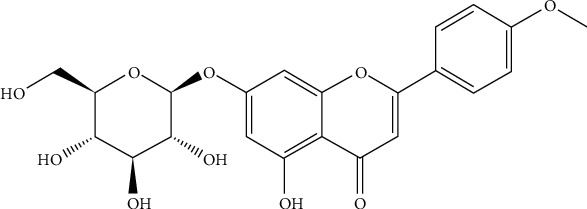
Chemical structure of tilianin. The molecular formula of tilianin is C_22_H_22_O_10_.

Tilianin, provided by the Xinjiang Institute of Materia Medica (Figure 1; Urumqi, China) was extracted from *Dracocephalum moldavica* L., reaching purity over 97% determined by high-performance liquid chromatography (HPLC) [14]. H9c2 cells were treated with tilianin at different concentrations for 4 h before and during the OGD/R process. H9c2 cells were randomly divided into two groups, with or without OGD/R procedure described as the above. These two cell groups were further divided into several subgroups receiving tilianin at different concentrations: 0 *μ*M, 0.8 *μ*M, 4.0 *μ*M, 20.0 *μ*M, and 100.0 *μ*M. Regarding the evaluation of the mechanism of action, cells were preincubated with 5 *μ*M KN93 (Sigma-Aldrich; St. Louis, MO, USA), a selective inhibitor of CaMKII, for 1 h prior to the treatment with tilianin, which was added after the replacement of the culture medium with a fresh one.

### 2.2. Cell Viability, Lactate Dehydrogenase Level, and Nuclear Staining Assay

Cell viability and the concentration of lactate dehydrogenase (LDH) release were detected using 3(4,5-dimethylthiazol-2-yl)-5-(3-carboxymethoxypheny-l)-2-(4-sulfophenyl)-2*H*-tetrazolium (MTS) and CytoTox-ONE homogeneous membrane integrity assay, respectively, following the manufacturer's instructions (Promega, Madison, WI, USA).

Changes in nuclear staining in H9c2 cells were evaluated by Hoechst 33342 (Dojindo Laboratory, Kumamoto, Japan). The staining degree was analyzed using a Cellomics ArrayScan V^TI^ High-Content Analysis (HCA) Reader (Thermo Fisher Scientific Cellomics, Waltham, MA, USA), and we selected target mean average fluorescent intensities (Mean_TargetAvgInten) as the evaluation values.

### 2.3. Molecule Docking Analysis

The molecular docking analysis was used to predict the structure of the binding between tilianin and CaMKII*δ* using computation methods. The crystal structure of the recombinant human CaMKII*δ* isoform complexed with SU6656 was downloaded at a resolution of 1.9 Å from the PDB database (PDB code: 2WEL), and the procedure was performed as previously described [16].

### 2.4. CaMKII Activity Assay

The substrate containing recombinant CaMKII*δ* (50 ng, Invitrogen) and autocamtide-2 peptide (KKALRRQETVDAL, 5 *μ*g) was incubated with the assay buffer, as described by our previous report [16]. The results were presented as the quantified relative ADP level and kinase activity.

### 2.5. Determination of Mitochondrial Membrane Potential and Superoxide Levels

The changes in intracellular mitochondrial function were measured by Rhodamine 123 (Rh123) (Dojindo Laboratory) and MitoSOX Red (Invitrogen) and detected using a Cellomics ArrayScan V^TI^ HCA Reader (Thermo Fisher Scientific Cellomics) as we previously described [19]. The experimental data were recorded as Mean_TargetAvgInten values.

### 2.6. Cellular Immunofluorescence

A cellular immunofluorescence assay was performed as previously described, and the fluorescence was read on a Cellomics ArrayScan V^TI^ HCA Reader (Thermo Fisher Scientific Cellomics) [20]. The primary and secondary antibodies used in this study are shown in Table 1. The translocation of p-JNK1/2, p-p65, p38 MAPK, and p-ERK1/2 was quantified as the Mean_CircRingAvgIntenDiff value. The nuclear fluorescence intensity of p-c-Jun and the cytosolic fluorescence intensity of *β*-tubulin, Bcl-2, Bax, cytochrome *c*, and CaMKII were represented as Mean_TargetAvgInten values.

### 2.7. ELISA Assay for Tumor Necrosis Factor Alpha (TNF-*α*) and Interleukin-6 (IL-6)

TNF-*α* and IL-6 concentration in H9c2 cell supernatant was evaluated using the respective ELISA kits (Invitrogen), following the manufacturer's instructions. The absorbance was read at 450 nm, and the concentration was expressed as pg/mL in the cell supernatant.

### 2.8. Animal Treatment and Experimental Protocols

Two-month-old male SD rats (300 ± 20 g) were purchased from the Beijing Vital River Laboratory Animal Technology Company (Beijing, China). Rats were housed in a pathogen-free environment under a temperature of 20-24°C and 12 h/12 h light/dark cycle. They were kept with free access to food and water for one week to acclimate to the housing environment. Animal experiments were approved by the ethical committee of the Institute of Medicinal Biotechnology, Beijing, China (approval number: IMB-201806-D8-02).

**Figure 2:**
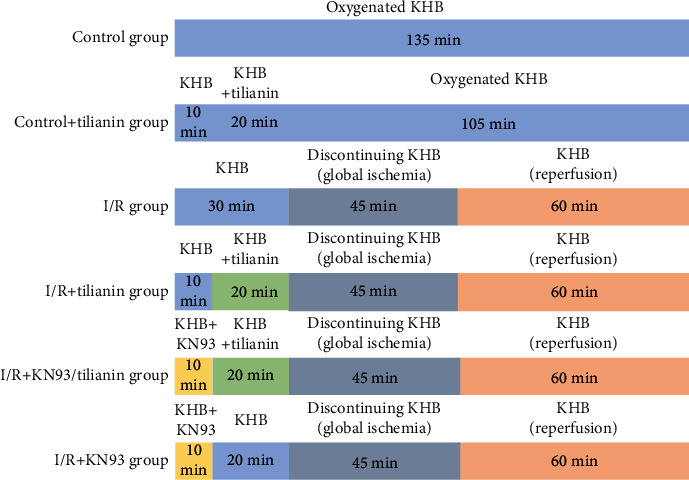
*Ex vivo* experimental procedure of the isolated rat hearts subjected to I/R and treated with tilianin and/or KN93. Isolated rat hearts were randomly divided into control, control+tilianin, I/R, I/R+tilianin, I/R+tilianin/KN93, and I/R+KN93 groups. The hearts of the control group were perfused with KHB for 30 min and then for the same period of time as the global I/R. The hearts of the control+tilianin group received 10 min perfusion with KHB and then 20 min KHB containing 4 *μ*M tilianin and continuous perfusion with oxygenated KHB for the same period of time as the control group. The hearts of the I/R group were subjected to global ischemia by interrupting KHB perfusion for 45 min, followed by reperfusion for 60 min. The hearts of the I/R+tilianin group were perfused with KHB containing 4 *μ*M tilianin for 20 min before I/R. The hearts of the I/R+KN93/tilianin group were perfused with 2.5 *μ*M KN93 for 10 min before tilianin perfusion. The hearts of the I/R+KN93 group were perfused with 2.5 *μ*M KN93 for 10 min and KHB for 20 min before I/R. After monitoring of the heart cardiodynamic parameters during I/R procedure, the isolated perfused rat heart was placed in ice-cold KHB for the subsequent measurement of western blot assay and other biochemical indicators.

An isolated heart ischemia/reperfusion model was established using the Langendorff apparatus (AD Instruments, Sydney, Australia) as previously reported [21, 22]. The isolated hearts were stabilized with oxygenated Krebs-Henseleit bicarbonate buffer (KHB) for a period of 30 min before being subjected to the following described experimental protocols. To determine the effect and potential target of tilianin on the recovery of MIRI, the hearts were randomly divided into six groups (*n* = 12 hearts per group) (Figure 2): (i) control group: the isolated hearts were perfused with KHB for 30 min and then for the same period of time as the global I/R; (ii) control+tilianin group: the isolated hearts received 10 min perfusion with KHB and then 20 min KHB containing 4 *μ*M tilianin and continuous perfusion with oxygenated KHB for the same period of time as the control group; (iii) I/R group: the isolated hearts were perfused with KHB for 30 min then subjected to 45 min global ischemia achieved by discontinuing the perfusion of KHB but with a subsequent reperfusion for 60 min; (iv) I/R+tilianin group: the isolated hearts received 10 min perfusion with KHB and then 20 min perfusion with KHB containing 4 *μ*M tilianin before I/R described above; (v) I/R+KN93/tilianin group: the isolated hearts in this group were perfused with KHB containing 2.5 *μ*M CaMKII inhibitor KN93 for 10 min, then with 4 *μ*M tilianin for the whole reperfusion period prior to I/R; and (vi) I/R+KN93 group: the isolated hearts were perfused with KHB containing 2.5 *μ*M CaMKII inhibitor KN93 for 10 min, then with 20 min KHB for the remaining reperfusion period prior to I/R. After monitoring of the heart cardiodynamic parameters during the I/R procedure, the isolated perfused rat heart was placed in ice-cold KHB for the measurement of western blot assay and other biochemical indicators.

### 2.9. Measurement of Heart Cardiodynamic Parameters

The following myocardial functional parameters were continuously monitored using a computer-based data acquisition system (AD Instruments, Sydney, Australia): left ventricular systolic pressure (LVSP), left ventricular end-diastolic pressure (LVEDP), left ventricular developed pressure (LVDP) equal to (LVSP-LVEDP), maximum rise/fall in velocity of left intraventricular pressure (+*d*p/*d*t_max_ and −*d*p/*d*t_min_), and heart rate (HR). Recovery in LVSP, LVDP, +*d*p/*d*t_max_, and −*d*p/*d*t_min_ was expressed as the percentage of the value 1 min before ischemia.

### 2.10. Measurement of Na^+^-K^+^-ATPase Activity and ATP Concentration

A tissue sample of the isolated working heart was lysed in a protein lysate buffer (pH 7.4, 50 mM Tris, 100 *μ*M EDTA, 0.25 M sucrose, 1% SDS, 1% NP40, 1 *μ*g/mL leupeptin, 1 *μ*g/mL pepstatin A, and 100 *μ*M phenylmethylsulfonyl fluoride (PMSF)), then homogenized. The measurement of Na^+^-K^+^-ATPase activity and adenosine triphosphate (ATP) level was performed using an adenylpyrophosphatase (ATPase) activity assay kit (MAK113 malachite green assay, Sigma-Aldrich) and a luciferase-based bioluminescence assay kit (Sigma-Aldrich), respectively, according to the manufacturer's instructions.

### 2.11. Western Blot Analysis

Total proteins from H9c2 cells and working heart tissues were extracted using RIPA lysis buffer, separated by electrophoresis on an SDS-polyacrylamide gel and transferred to a polyvinylidene difluoride (PVDF) membrane (Millipore, Billerica, MS, USA). The membrane was blocked with 5% nonfat milk in Tris-buffered saline/Tween 20 (TBST) and was incubated overnight with the following primary antibodies: anti-ox-CaMKII*δ* (Met281/282) (1 : 1000, GeneTex), anti-p-CaMKII*δ* (Thr286) (1 : 1000, Abcam), anti-CaMKII*α* (1 : 10000, Abcam), anti-CaMKII*β* (1 : 500, Abcam), anti-CaMKII*δ* (1 : 1000, Abcam), anti-CaMKII*γ* (1 : 500, Abcam), anti-p-JNK1/2 (T183/Y185) (1 : 1000, CST), anti-JNK1/2 (1 : 1000, CST), anti-p-NF-*κ*B p65 (Ser536) (1 : 1000, Abcam), anti-NF-*κ*B p65 (1 : 1000, Abcam), anti-Bcl-2 (1 : 500, Abcam), anti-Bax (1 : 500, Abcam), anti-cytochrome *c* (1 : 1000, Abcam), and anti-GAPDH (1 : 1000, ZSGB-Bio, Beijing, China). The membrane was then washed and probed with horseradish peroxidase-conjugated goat anti-rabbit or anti-mouse antibody (1 : 8000, ZSGB-Bio) to enhance the chemiluminescence that was detected using a Fusion-FX6 imaging system (Vilber Lourmat, Marne-la-Valle, France).

### 2.12. Caspase-3 and Caspase-9 Activity Detection

Caspase-3 and caspase-9 activity in H9c2 cell supernatant and isolated heart was measured by the Caspase-Glo 3 and Caspase-Glo 9 assay kits (Promega) according to the manufacturer's instructions.

### 2.13. Statistical Analysis

GraphPad Prism version 6.0 (GraphPad Inc., La Jolla, CA, USA) was used to perform the statistical analysis. Results are presented as the mean ± standard error of the mean (S.E.M.) or mean ± standard error (S.D.). Comparisons were performed using Student's *t*-test or one-way ANOVA followed by Tukey's multiple comparison test or Dunnett's test. *P* < 0.05 was considered statistically significant.

## 3. Results

### 3.1. Tilianin Treatment Protects H9c2 Cardiomyocytes against OGD/R-Induced Cytotoxicity

**Figure 3:**
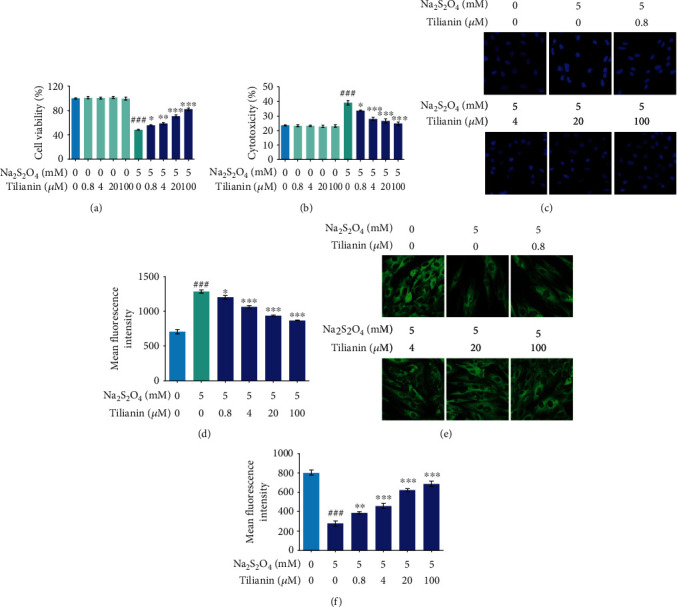
Tilianin treatment protects H9c2 cardiomyocytes against OGD/R-induced cytotoxicity. (a) Tilianin increased cell viability as evaluated by MTS assay. (b) Tilianin decreased the release of lactate dehydrogenase (LDH) after OGD/R injury. (c) Representative images of nuclei stained with Hoechst 33342 (20×). (d) Tilianin decreased the mean fluorescence intensity of nuclei stained with Hoechst in H9c2 cells after OGD/R injury. (e) Representative images of the microtubule morphology stained with *β*-tubulin (20×). (f) Tilianin increased mean fluorescence intensity of *β*-tubulin in H9c2 cells after OGD/R injury. Results are expressed as the mean ± S.E.M.*n* = 6. ^###^*P* < 0.001*vs.* control, ^∗^*P* < 0.05, ^∗∗^*P* < 0.01, and ^∗∗∗^*P* < 0.001*vs.* OGD/R.

The protective effect of tilianin on OGD/R-treated H9c2 cells was examined using three cytotoxicity assays and one microtubule morphology test. The results of the MTS assay demonstrated that cell viability after OGD/R injury significantly decreased compared to that of the control group (Figure 3(a), *P* < 0.001) and LDH release was much higher in the OGD/R group than that in the control group (Figure 3(b), *P* < 0.001). H9c2 cells injured by OGD/R showed condensed nuclei with strong fluorescence intensity (Figures 3(c) and 3(d), *P* < 0.001) and the disrupted microtubules with weak fluorescence intensity (Figures 3(e) and 3(f), *P* < 0.001). However, tilianin treatment following OGD/R injury significantly increased H9c2 cell viability at 0.8 *μ*M, 4.0 *μ*M, 20.0 *μ*M, and 100.0 *μ*M in a dose-dependent manner (Figure 3(a), *P* < 0.05‐0.001). Similarly, tilianin treatment at 0.8 *μ*M, 4.0 *μ*M, 20.0 *μ*M, and 100.0 *μ*M resulted in a dose-dependent reduction in LDH release in OGD/R-injured cells (Figure 3(b), *P* < 0.05‐0.001) and reduced the cytotoxic effects as demonstrated by the reduced nuclear condensation and fluorescence intensity using Hoechst staining (Figures 3(c) and 3(d), *P* < 0.05‐0.001). Besides, the microtubule morphology was ameliorated by tilianin, as demonstrated by the relative intact microtubule structure and stronger fluorescence intensity compared with the OGD/R group (Figures 3(e) and 3(f), *P* < 0.01‐0.001). Thus, these results indicated that tilianin decreased the impact of OGD/R-induced injury in H9c2 cells. Besides, no differences were observed in cell viability between control cells and tilianin-treated cells, indicating no toxicity under the conditions used.

### 3.2. Prediction of CaMKII*δ*/Tilianin Binding Ability

**Figure 4:**
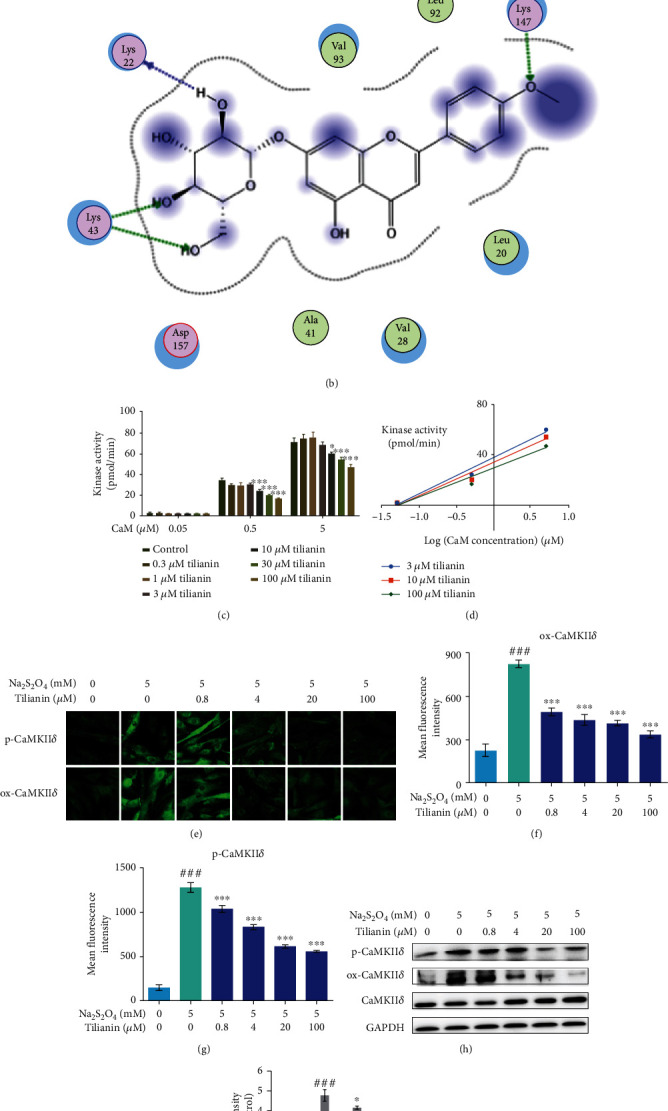
Prediction of CaMKII*δ*/tilianin binding ability and inhibitory effect of tilianin on CaMKII*δ* kinase activity. (a) Three-dimensional model of tilianin binding with the binding domain of CaMKII*δ*. (b) Two-dimensional ligand interaction diagram of tilianin and CaMKII*δ*. (c) Inhibitory effect of tilianin on CaMKII*δ* kinase activity *in vitro* (*n* = 5). (d) Double-reciprocal analysis of the inhibitory effect. (e) Representative images of p-CaMKII*δ* and ox-CaMKII*δ* staining (20×). (f, g) Mean fluorescence intensity of p-CaMKII*δ* and ox-CaMKII*δ* (*n* = 6). (h) Representative western blot bands of the expression of p-CaMKII*δ* and ox-CaMKII*δ* in H9c2 cells. (i) Quantitative analysis indicated that tilianin inhibited the p-CaMKII*δ*/CaMKII*δ* and ox-CaMKII*δ*/CaMKII*δ* ratios (*n* = 4). Results are expressed as the mean ± S.E.M.^###^*P* < 0.001*vs.* control, ^∗^*P* < 0.05, ^∗∗^*P* < 0.01, and ^∗∗∗^*P* < 0.001*vs.* OGD/R.

As a critical signaling node in promoting cell apoptosis, CaMKII causes mitochondrion-associated cardiomyocyte apoptosis during MIRI [23]. The western blot assay showed that CaMKII*δ* expression in H9c2 cells was higher than the one of the other three isoforms (Supplementary Figure 1). Therefore, CaMKII*δ* was chosen to explore the relationship between tilianin and CaMKII against MIRI to perform our subsequent experiments. The results of the molecular docking analysis demonstrated that tilianin bound CaMKII*δ* with a favorable binding score of -23.3 kcal/mol. Furthermore, tilianin formed four hydrogen bond interactions with Lys43, Lys22, and Lys147 in CaMKII*δ*. The glucoside group of tilianin formed three hydrogen bond interactions with the nitrogen atom of Lys43 and the oxygen atom of Lys22. In addition, the molecular core region of tilianin interacted with the active site of CaMKII*δ* formed by the residues Leu20, Val28, Ala41, Leu92, and Val93 via van der Waals/hydrophobic interactions (Figures 4(a) and 4(b)).

### 3.3. Tilianin Inhibits CaMKII*δ* Kinase Activity in a Limited Ability But Suppresses the Expression of CaMKII*δ* against OGD/R-Induced Toxicity in H9c2 Cardiomyocytes

An *in vitro* kinase assay was performed to explore the functional effect of tilianin binding on CaMKII*δ* kinase activity. The results showed that CaMKII*δ* kinase activity was significantly blocked by relatively higher concentrations of tilianin (10 *μ*M to 100 *μ*M) (Figure 4(c), *P* < 0.05 and 0.001). Double-reciprocal analysis of these data did not yield straight lines intersecting on the *y*-axis (Figure 4(d)), indicating noncompetitive and nonspecific inhibition of CaMKII*δ* by tilianin concerning the Ca^2+^/CaM complex.

In addition to the evaluation of CaMKII*δ* activation by binding to Ca^2+^/CaM, this enzyme can be activated by cellular oxidized and phosphorylated groups that result in the exacerbation of mitochondrion-associated programmed cell death during acute I/R [8]. The expression of oxidized (ox-) and phosphorylated (p-) CaMKII*δ* was detected by both visual immunofluorescence and western blotting assay on OGD/R-injured H9c2 cells. As shown in Figures 4(e)–4(i), the ox-CaMKII and p-CaMKII were increased due to OGD/R-induced injury in H9c2 cells, as indicated by the stronger fluorescence intensity and higher ratio of ox-CaMKII/CaMKII and p-CaMKII/CaMKII as compared to the control cells (all *P* < 0.001). Tilianin treatment in the concentration range of 0.4 *μ*M to 100.0 *μ*M reduced ox-CaMKII and p-CaMKII expression in OGD/R-injured cells in a dose-dependent manner, as shown by both immunofluorescence and western blotting (Figures 4(e)–4(i), *P* < 0.05‐0.001). Combined with the results on the interaction between tilianin and CaMKII, these results suggested that the cardioprotection mediated by tilianin against OGD/R injury might be primarily due to the inhibitory effects on ox-CaMKII and p-CaMKII.

### 3.4. Tilianin Exerts Cardioprotection due to OGD/R Injury through Inhibiting CaMKII-Mediated Mitochondrial Dysfunction, Apoptosis, and Inflammation

**Figure 5:**
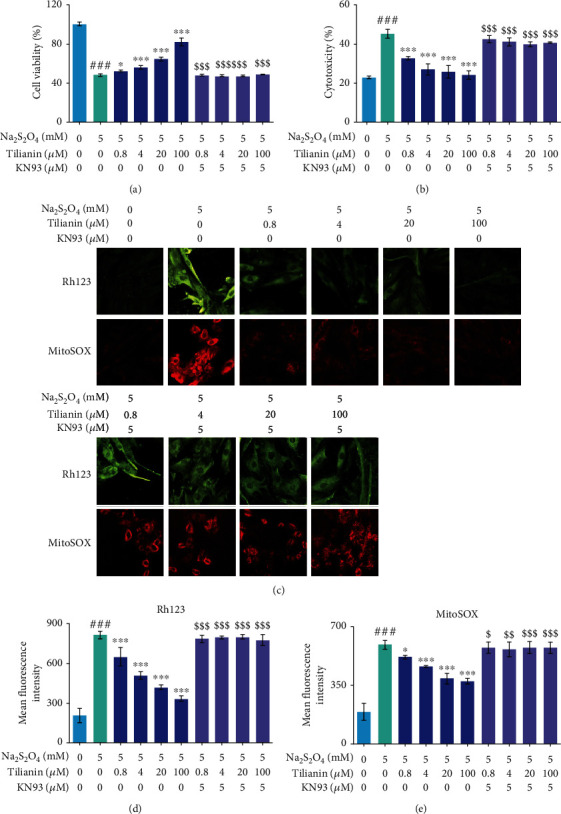
Tilianin exerts a protective effect against OGD/R injury through mitochondrion preservation via CaMKII inhibition. (a) The effect of tilianin treatment on increasing cell viability against OGD/R-induced toxicity was blocked by CaMKII pharmacological inhibition using KN93. (b) The effect of tilianin treatment on decreasing LDH release on OGD/R-injured H9c2 cells was reduced due to KN93 treatment. (c) Representative images of MMP staining by Rh123 (20×) and superoxide by MitoSOX in mitochondria (20x). (d, e) The protective effect of tilianin on mitochondrial function evaluated by Rh123 staining (d) and MitoSOX staining (e) was abolished when OGD/R-injured H9c2 cells were pretreated with KN93. Results are expressed as the mean ± S.E.M.*n* = 6. ^###^*P* < 0.001*vs.* control, ^∗^*P* < 0.05, ^∗∗∗^*P* < 0.001*vs.* OGD/R; ^$^*P* < 0.05, ^$$^*P* < 0.01, and ^$$$^*P* < 0.001*vs.* OGD/R+tilianin.

As shown in Figures 5(a) and 5(b), after CaMKII inhibition with KN93, the ability of tilianin to save the decreased cell viability and reduce the increased membrane leakage after OGD/R injury was significantly blocked at any of its tested concentrations (all *P* < 0.001).

**Figure 6:**
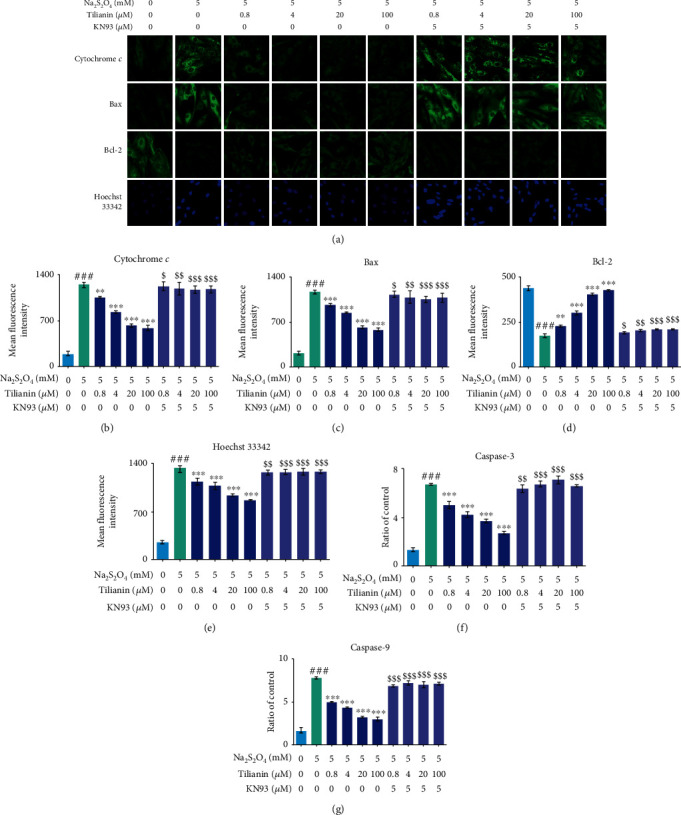
Tilianin protects H9c2 cardiomyocytes against OGD/R injury through mitochondrion-associated apoptotic signaling via CaMKII inhibition. (a) Representative images of cytochrome *c*, Bax, and Bcl-2 expression by immunohistochemistry and nuclear changes stained by Hoechst 33342 (20×). (b–e) Restorative effects of tilianin treatment on the expression of cytochrome *c*, Bax, and Bcl-2 and nucleus damage caused by OGD/R-induced toxicity were abolished by KN93. (f, g) The inhibitory effect on caspase-3/9 activity by tilianin was reduced due to KN93 treatment. Results are expressed as the mean ± S.E.M.*n* = 6. ^###^*P* < 0.001*vs.* control, ^∗∗^*P* < 0.01, ^∗∗∗^*P* < 0.001*vs.* OGD/R; ^$^*P* < 0.05, ^$$^*P* < 0.01, and ^$$$^*P* < 0.001*vs.* OGD/R+tilianin.

In addition to the myocardial cytotoxicity assay, mitochondrion-targeted cardioprotection by the tilianin treatment against OGD/R damage was evaluated, which revealed a concentration-dependent improvement in mitochondrial membrane potential (MMP) and suppression in the generation of mtROS at 0.8 *μ*M, 4.0 *μ*M, 20.0 *μ*M, and 100.0 *μ*M (Figures 5(c)–5(e), *P* < 0.05‐0.001). The increase of cytochrome *c* and Bax expression, activation of caspase-9 and caspase-3, and the decrease of Bcl-2 expression in H9c2 cells when subjected to OGD/R were prevented by tilianin treatment (Figures 6(a)–6(d), 6(f), and 6(g), *P* < 0.01‐0.001). However, after preincubation with KN93, the benefits of tilianin on protecting the mitochondrion, involving the enhancement of MMPs and decrease of mtROS as well as the beneficial regulation of proteins associated with apoptosis such as Bax, cytochrome *c*, Bcl-2, and caspase-9 and caspase-3, were significantly reduced (Figures 5(c)–5(e), 6(a)–6(d), 6(f), and 6(g), *P* < 0.05‐0.001). Besides, Hoechst staining also showed that tilianin reduced nuclear condensation and consequent brightness, but these effects were blocked by KN93 (Figures 6(a) and 6(e), *P* < 0.01‐0.001). These results demonstrated that tilianin might have a specific effect on the amelioration of mitochondrion-mediated apoptosis via inhibiting CaMKII, thus against OGD/R injury.

**Figure 7:**
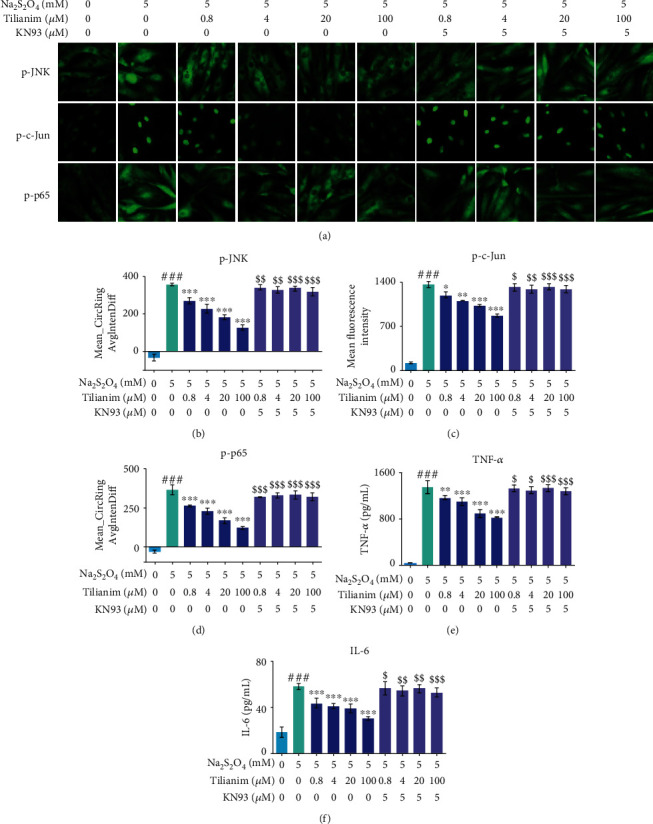
Tilianin protects H9c2 cardiomyocytes against OGD/R injury through JNK/NF-*κ*B-related inflammatory suppression via CaMKII inhibition. (a) Representative immunohistochemical images of p-JNK, p-c-Jun, and p-p65 expression (20×). (b–d) Ameliorative effects of tilianin on JNK/c-Jun and p-p65 activation were reduced by KN93. (e, f) Inhibitory effects on TNF-*α* and IL-6 release by tilianin were prevented by KN93 treatment. Results are expressed as the mean ± S.E.M.*n* = 6. ^###^*P* < 0.001*vs.* control, ^∗^*P* < 0.05, ^∗∗^*P* < 0.01, and ^∗∗∗^*P* < 0.001*vs.* OGD/R; ^$^*P* < 0.05, ^$$^*P* < 0.01, ^$$$^*P* < 0.001*vs.* OGD/R+tilianin.

The activation of the MAPK members and overlapping NF-*κ*B-related inflammatory reactions also contribute to cell apoptosis [24]. The activation of JNK/c-Jun and NF-*κ*B-mediated inflammation were both decreased by tilianin in OGD/R-injured H9c2 cells (Supplementary Figures 2A, D-H and Figures 7(a)–7(f), *P* < 0.05‐0.001), while the activation of ERK1/2 and p38 MAPK was not affected by tilianin at any detected concentration (Supplementary Figures 2A-C). In response to CaMKII inhibition by KN93, tilianin could not exert its effects on the nuclear translocation blockage of p-JNK and p-p65, the decrease of p-c-Jun expression, and the reduction in the release of proinflammatory factors such as TNF-*α* and IL-6 at concentrations from 0.4 *μ*M to 100.0 *μ*M under OGD/R conditions (Figures 7(a)–7(f), *P* < 0.05‐0.001). Therefore, these results suggested that tilianin might exert an effect on reducing inflammatory response caused by JNK/c-Jun and NF-*κ*B through CaMKII activation under OGD/R-generated deficits.

### 3.5. Tilianin Protects I/R-Injured Rat Heart Tissue via CaMKII Inhibition

**Figure 8:**
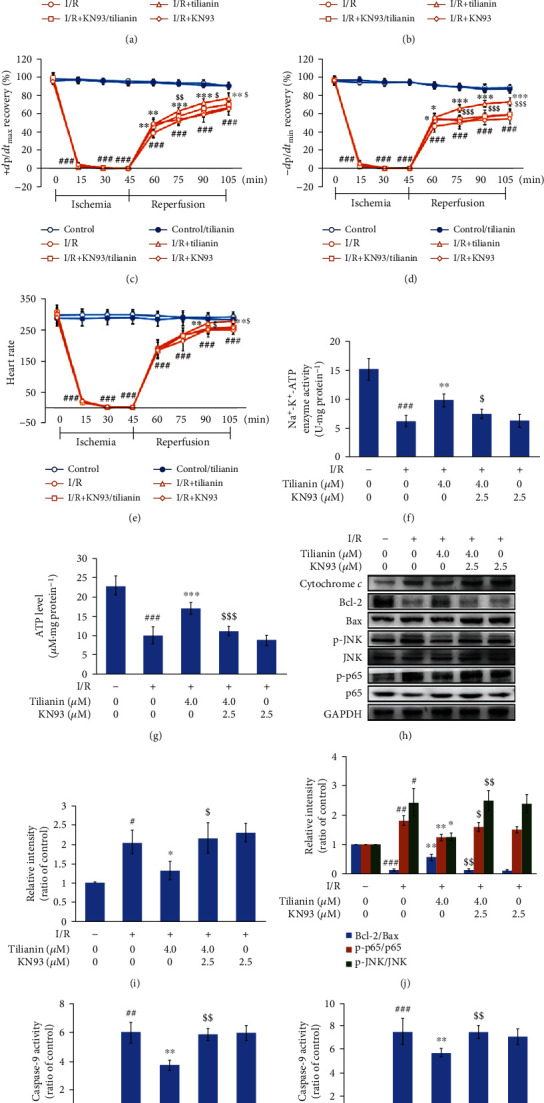
CaMKII contributes to tilianin protection against ischemia/reperfusion (I/R) injury in heart tissue. (a–e) LVSP (a), LVDP (b), +*d*p/*d*t_max_ (c), −*d*p/*d*t_min_ (d), and heart rate (e) values recovered after tilianin treatment, effects that were prevented when the tissue was pretreated with KN93 (*n* = 12). (f, g) Na^+^-K^+^-ATPase activity and ATP concentration increased following tilianin treatment but weakened after pretreatment with KN93 (*n* = 5). (h) Representative immunoblots illustrating the expression of cytochrome *c*, Bax, Bcl-2, p-JNK, JNK, p-p65, and p65 (*n* = 5). (i, j) Quantitative analysis indicating that restoration of the cytochrome *c* expression and Bcl-2/Bax, p-JNK/JNK, and p-p65/p65 ratios by tilianin was reversed by pretreatment with KN93. (k, l) Inhibitory effect on caspase-9 (k) and caspase-3 (l) activity by tilianin blocked by KN93 pretreatment (*n* = 5). Results are expressed as the mean ± S.D.^#^*P* < 0.05, ^##^*P* < 0.01, and ^###^*P* < 0.001*vs.* control; ^∗^*P* < 0.05, ^∗∗^*P* < 0.01, ^∗∗∗^*P* < 0.001*vs.* I/R; ^$^*P* < 0.05, ^$$^*P* < 0.01, ^$$$^*P* < 0.001*vs.* I/R+tilianin.

To further establish that CaMKII inhibition is responsible for the cardioprotection, isolated working hearts were perfused with tilianin and KN93 prior to ischemia. The working hearts did not show any changes in the value of cardiodynamic parameters after 4 *μ*M tilianin treatment compared with control hearts (Figures 8(a)–8(e)). Perfusion with tilianin at 4 *μ*M for 20 min resulted in functional recovery of the I/R hearts, including an increase in the recovery of LVSP, LVDP, ±*d*p/*d*t_max/min_, and heart rate throughout the reperfusion period (Figures 8(a)–8(e), *P* < 0.05‐0.001), while these effects were clearly arrested by the addition of the CaMKII inhibitor KN93 at 2.5 *μ*M (*P* < 0.05‐0.001). As no differences were observed in these detected cardiodynamic parameters between control hearts and control hearts treated with tilianin, the hearts of the control group, I/R group, and I/R group treated with tilianin and/or KN93 were used for subsequent biochemical indicator and signaling molecule detection.

Protection of mitochondrial activity, indicated by Na^+^-K^+^-ATPase activity and ATP concentration, exerted by tilianin (Figures 8(f) and 8(g), *P* < 0.05 and 0.001) was also attenuated when KN93 pretreatment was performed (*P* < 0.05 and 0.001). In the mitochondrion-mediated signaling pathway, the favorable alteration in the expression of cytochrome *c*, the ratio of proapoptotic Bax and antiapoptotic Bcl-2, and subsequent activity of caspase-9 and caspase-3 induced by I/R following treatment with tilianin (Figures 8(h)–8(l), *P* < 0.05-0.01) were reversed after pharmacological CaMKII inhibition (*P* < 0.05-0.01). Similarly, the suppressive effects of tilianin on the JNK/NF-*κ*B signaling pathway, indicated by the value of p-JNK/JNK and p-p65/p65, were weakened after CaMKII inhibition (Figures 8(h) and 8(j), *P* < 0.05 and 0.01).

In addition, isolated working hearts subjected to I/R injury and pretreated with 2.5 *μ*M KN93 alone with a 20 min interval showed a slight amelioration at the early period of the reperfusion stage (60 min or 75 min reperfusion time), compared with the hearts of the I/R group (Figures 8(a)–8(e)), but without beneficial effects with a longer reperfusion period and without an ameliorative effect on the mitochondrion-dependent apoptosis and JNK/NF-*κ*B signaling pathway (Figures 8(f)–8(j)), indicating that single CaMKII inhibition by KN93 for a certain time prior to I/R could not completely protect cardiac function from I/R injury. Collectively, the CaMKII-related mitochondrial apoptotic pathway and JNK/NF-*κ*B cascade might be involved in the protective effects of tilianin on I/R hearts.

## 4. Discussion

The present study is aimed at verifying the involvement and function of CaMKII on the mitochondrion-associated apoptotic pathway and MAPK/NF-*κ*B-related inflammatory reaction in the protection exerted by tilianin against MIRI. Two major contributions were demonstrated in this study. First, tilianin was identified as a potential CaMKII inhibitor because it prevented I/R-induced myocardial dysfunction both *in vitro* and *ex vivo*. Second, a cellular signaling profile of tilianin in CaMKII inhibition was provided, characterized by the suppression of CaMKII-dependent signaling of the mitochondrion-associated apoptotic pathway and JNK/NF-*κ*B-triggered inflammatory reaction. These findings provided original evidence and understanding of the potential therapeutic mechanism associated with the cardioprotective effects of tilianin against MIRI.

MIRI is an aggravated injury caused by reperfusion after the restoration of the blood flow following cardiac surgery and myocardial infarction, resulting in several potential deteriorating conditions including heart failure, malignant arrhythmia, and sudden death [25]. In our study, an OGD/R-induced toxicity model was established in H9c2 cardiomyocytes and an I/R injury was performed in isolated hearts to establish conditions that mimic MIRI. Prior to the identification of the anti-MIRI effects of tilianin, the safe dosage was evaluated as ranging from 0.8 *μ*M to 100.0 *μ*M *in vitro* and at 4 *μ*M *ex vivo* in isolated working hearts prior to and during the MIRI process. Tilianin at these concentrations did not show any cytotoxicity on cardiomyocytes or cardiac injury of working hearts; however, tilianin exerted recovery of OGD/R-injured cardiomyocytes and maintained cardiac function in I/R-injured hearts due to MIRI. The protective effects of tilianin involved preservation of cell viability, membrane integrity, nuclear uniformity, and microtubule morphology, in addition to the improvement in cardiac hemodynamic performance, consistent with previous studies which demonstrated that tilianin exhibited anti-ischemic and antihypoxic effects in I/R rats [12, 14]. Thus, tilianin exerted a protective effect on cardiac performance under MIRI, but not under physiological conditions.

The same as many flavonoids, tilianin exerts an antioxidant activity contributing to its cardioprotective effects. However, redox balance was unlikely to be the sole mechanism underlying its cardioprotective effect. First, the burst of ROS was far in excess of the radical scavenging capacity at the tested concentrations of tilianin, which was supported by the observation of its limited antioxidative effects. Indeed, although the generation of mtROS *in vitro* was fully inhibited, the cardiac SOD and GSH-Px activity did not fully increase in the I/R *ex vivo* model (Supplementary Figures3A and 3B). Second, tilianin had an effect on the changes of a broad range of cellular stresses involving anti-apoptotic and anti-inflammatory inhibition, which were not thought to directly contribute to free radical generation [26, 27]. In view of these results underlining that the redox balance was not the pivotal mechanisms contributing to tilianin-mediated cardioprotection, tilianin might act on other mechanisms involved in MIRI pathology.

CaMKII, a pleiotropic signal that regulates cardiomyocyte contractility, inflammation, metabolism, gene expression, and cell survival [28], acts as a crucial regulatory mediator in multiple cellular pathways including mitochondrion-related apoptotic cascades and MAPK-mediated inflammation. During the resting state, CaMKII tends to be inactive. When a large amount of Ca^2+^ influx penetrates into cells, the CaMKII holoenzyme is stimulated to activate the conformation change, while Ca^2+^/CaM combines with regulatory domains, allowing the substrate access to catalytic sites. Once active, high levels of ROS are available for various posttranslational modifications that convert CaMKII from a Ca^2+^/CaM-activated enzyme to a Ca^2+^/CaM autonomous enzyme [3]. These Ca^2+^/CaM-independent forms of CaMKII, due to threonine-286 (autophosphorylation) and methionine-281/282 (oxidation), are associated with I/R-induced myocardial apoptosis [3, 29, 30]. In the progress of MIRI, the phosphorylation of Thr286 site and oxidation of Met281/282 site activate cytosolic CaMKII, leading to mitochondrial dysfunction, which in turn triggers mitochondrion-mediated intrinsic apoptotic events [31].

The docking analysis was used to explore the potential interaction between tilianin and CaMKII*δ*, demonstrating that CaMKII*δ* could interact with tilianin with a favorable binding score, accompanied by interactions through van der Waals forces and hydrophobic bonding. According to the affinity of small molecules, tilianin had a mild suppression effect (IC50 over 100 *μ*M) compared to the inhibition effect of KN93 *in vitro* (IC50 6.37 *μ*M, Supplementary Figure 3C). Importantly, tilianin treatment clearly inhibited OGD/R-increased expression of ox-CaMKII and p-CaMKII. In particular, under conditions that specifically inhibited CaMKII by KN93, tilianin-mediated cardioprotection against MIRI was prominently abolished both *in vitro* and *ex vivo*, as shown by the reduction of cell viability and the deterioration of cardiac hemodynamic performance. After considering these observations, we speculated that, due to the different activation sites on CaMKII, the cardioprotection exerted by tilianin against MIRI might be due to the modulation of both ox-CaMKII and p-CaMKII activation and intervention in their interrelated signaling pathways.

Evidence indicates that excessive stimulation of mitochondrial CaMKII may be a mechanism for myocardial dysfunction or apoptosis during MIRI [7]. When CaMKII is overactivated in cultured cardiomyocytes, apoptotic pathways are triggered mainly due to oxidative stress, along with large Ca^2+^ influx into cells and enhanced release of cytochrome *c* from the mitochondrion [32]. Afterward, whether the cells survive or undergo apoptosis depends on the balance between up- and down-regulation of anti-apoptotic and pro-apoptotic proteins. Previous studies showed that the effects of tilianin against MIRI included mitochondrion-related events through the improvement of mitochondrial structural integrity, inhibition of the opening of the mitochondrial permeability transition pore (mPTP), and reduced expression of cytochrome *c*, Bax, and apoptosis-inducing factor (AIF) in the cytosol [13, 14]. Consistent with these studies, our study supported the improvement exerted by tilianin on the mitochondrial status, specifically on the increase of MMP and reduction of mtROS in H9c2 cardiomyocytes subjected to OGD/R, as well as the amelioration of the mitochondrial activity such as the decrease of Na^+^-K^+^-ATPase activity and increase of ATP concentration of isolated working hearts injured by I/R. Moreover, tilianin inhibited the release of the pro-apoptotic cytochrome *c*, maintained the equilibrium between Bcl-2 and Bax, decreased the activation of caspase-9 and caspase-3, and ultimately reduced DNA damage caused by the disruption of MMP due to MIRI both *in vitro* and *ex vivo*. When the signaling balance preserved by tilianin was blocked by KN93, the mitochondrial and anti-apoptotic effects of tilianin treatment against MIRI were abolished, suggesting a close correlation between CaMKII and the action of tilianin on mitochondrion-associated apoptosis.

CaMKII is a key upstream kinase that transfers signals to JNK and/or p38 MAPK [33–35]. Regarding our results, tilianin altered the activation of the JNK signaling pathway but not the activation of p38 MAPK following OGD/R injury *in vitro*, and these effects on JNK/c-Jun of tilianin were all blocked in the presence of KN93 both *in vitro* and *ex vivo*, suggesting that JNK might be associated with the inhibitory effect of tilianin on CaMKII. It is known that active CaMKII directly targets ASK1, a MAPKKK capable of dual activation of the JNK pathway [36, 37], which places CaMKII upstream of JNK in the case of MIRI. Moreover, JNK activation plays an important role in apoptosis in response to a variety of stress signals, mediating protein phosphorylation of the Bcl-2 family to induce the apoptotic signaling pathways [38, 39]. Combined with the action of tilianin in mitochondrion-dependent cardiomyocyte apoptosis and the inhibition exerted by KN93, our hypothesis was that tilianin might inhibit MIRI-induced JNK activation through CaMKII in cardiomyocytes.

The activation of JNK triggers the activation of NF-*κ*B, which in turn induces the overproduction of proinflammatory cytokines, followed by an inflammatory response [23]. Our results demonstrated that tilianin-mediated cardioprotection was accompanied by a significant reduction in JNK/c-Jun signaling and subsequent NF-*κ*B inactivation both *in vitro* and *ex vivo*, accompanied by the removal of the inhibiting effect of KN93 on CaMKII. This is consistent with the discovery that downstream JNK of CaMKII might contribute to the pathology of inflammation and apoptosis in cardiomyocytes. Moreover, other investigators reported that tilianin exerts protective effects by inhibiting NF-*κ*B- and JNK-related pathways in different experimental models [15, 16, 40, 41]. Thus, considering the interaction between CaMKII and JNK/NF-*κ*B mentioned above, the mechanisms used by tilianin to exert cardioprotection, such as suppression of mitochondrion-mediated apoptosis and inflammation, might be dependent on the suppression of JNK/NF-*κ*B signaling by interfering with the action of CaMKII.

Accumulating evidence illustrates that KN93 may be beneficial in MIRI pathology [42–44]; however, an evident controversy exists regarding KN93 effects detected in different I/R models [45]. In our study, a limited protective effect of KN93 against I/R injury was found in isolated working hearts at the beginning of the perfusion when KN93 was perfused for 10 min with a 20-minute interval in advance. Since the specific inhibition of CaMKII by KN93 decreases Thr17 phosphorylation of CaMKII that is associated with an impairment of myocardial relaxation [45], our results were in accordance with the notion that the status of CaMKII phosphorylation seems unlikely to determine the degree of postischemic recovery of myocardial function [46].

Despite the results obtained, some limitations should be addressed in this work. First, as there is a large difference between the external and internal environment in the heart in an *ex vivo* condition, as well as the lack of normal physiological regulation of the heart function during the establishment of a Langendorff isolated heart perfusion model, the potential proposed regulatory mechanisms of tilianin for cardioprotection need to be further explored *in vivo* in an appropriate animal model. Second, although KN93 inhibitor was used in this study to investigate the relationship between tilianin and CaMKII and the consequent mechanism of action, CaMKII siRNA might be a more appropriate choice to avoid the off-target effects of tilianin. Third, the specific downstream signaling pathway of CaMKII targeted by tilianin such as JNK and NF-*κ*B needs to be further verified.

**Figure 9:**
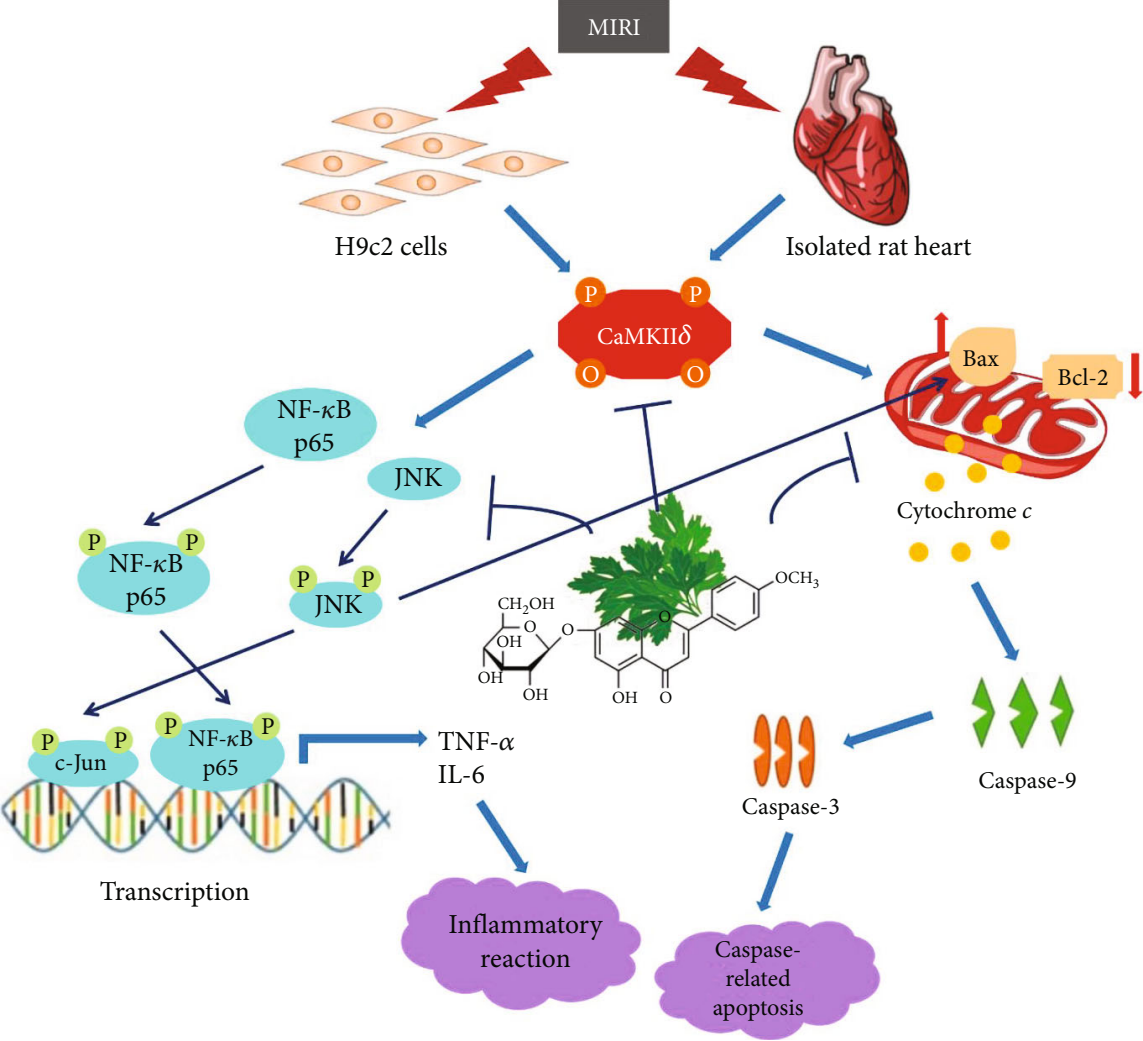
Tilianin exerts myocardial protection against I/R injury through its action on mitochondrion-related apoptosis and JNK/NF-*κ*B-activated inflammation via CaMKII. Bax: Bcl-2-associated X protein; Bcl-2: B cell lymphoma-2; CaMKII: Ca^2+^/calmodulin-dependent protein kinase II; IL-6: interleukin-6; JNK: c-Jun N-terminal protein kinase; NF-*κ*B: nuclear factor kappa-B; TNF-*α*: tumor necrosis factor alpha.

In conclusion, the present study characterized the underlying mechanisms of action of tilianin against MIRI, suggesting that tilianin might prevent mitochondrion-related apoptosis and JNK/NF-*κ*B-activated inflammation via CaMKII inhibition in the condition of cellular stress during I/R injury (Figure 9). These evidences highlighted the potential therapeutic benefits by reducing CaMKII signaling using tilianin during myocardial I/R.

## Figures and Tables

**Table 1:** Primary antibodies and secondary antibodies used in the cellular immunofluorescence assay.

Primary antibody	Dilution	Source	Secondary antibody (dilution, source)
Anti-oxidized-CaMKII (Met281/282) rabbit pAb	1 : 50	GeneTex	Alexa Fluor 546 goat anti-rabbit (1 : 1000, Invitrogen)
Anti-phospho-CaMKII (Thr286) rabbit pAb	1 : 200	Abcam	Alexa Fluor 546 goat anti-rabbit (1 : 1000, Invitrogen)
Anti-Bcl-2 rabbit mAb	1 : 200	Abcam	Alexa Fluor 488 goat anti-rabbit (1 : 1000, Invitrogen)
Anti-Bax rabbit mAb	1 : 100	Abcam	Alexa Fluor 488 goat anti-rabbit (1 : 1000, Invitrogen)
Anti-cytochrome *c* rabbit mAb	1 : 100	Abcam	Alexa Fluor 488 goat anti-rabbit (1 : 1000, Invitrogen)
Anti-phospho-SAPK/JNK (Thr183/Tyr185) rabbit mAb	1 : 200	CST	Alexa Fluor 546 goat anti-rabbit (1 : 1000, Invitrogen)
Anti-phospho-p38 MAPK (Thr180/Tyr182) rabbit mAb	1 : 200	CST	Alexa Fluor 546 goat anti-rabbit (1 : 1000, Invitrogen)
Anti-phospho-p44/42 MAPK (Thr202/Tyr204) mouse mAb	1 : 200	CST	Alexa Fluor 546 goat anti-mouse (1 : 1000, Invitrogen)
Anti-phospho-c-Jun (Ser63) rabbit mAb	1 : 100	Abcam	Alexa Fluor 546 goat anti-rabbit (1 : 1000, Invitrogen)
Anti-phospho-NF-*κ*B p65 (Ser536) rabbit pAb	1 : 100	Abcam	Alexa Fluor 546 goat anti-rabbit (1 : 1000, Invitrogen)
Anti-beta tubulin rabbit pAb	1 : 200	Abcam	Alexa Fluor 546 goat anti-rabbit (1 : 1000, Invitrogen)

Note: GeneTex, Irvine, CA, USA; CST: Cell Signaling Technology, Danvers, MA, USA; Abcam, Cambridge, MA, USA.

## Data Availability

The data used to support the findings of this study are available from the corresponding authors upon request.

## Supplementary Materials

Supplementary Materials Supplementary Figure 1: protein expression of CaMKII isoforms in H9c2 cardiomyocytes. Cell lysates were analyzed for CaMKII*α* (A), CaMKII*β* (B), CaMKII*δ* (C), and CaMKII*γ* (D) expression by western blotting using the specific antibodies. GAPDH expression was used for comparison. Supplementary Figure 2: tilianin decreases JNK/c-Jun and NF-*κ*B inflammatory signaling in OGD/R-injured H9c2 cardiomyocytes. (A) Representative images of p-p38, p-ERK1/2, p-JNK, p-c-Jun, and p-p65 expression (20x). (B-E) Mean_CircRingAvgIntenDiff values that describe the translocation of cytosolic p-p38 (B), p-ERK1/2 (C), p-JNK (D), and p-p65 (E) to the nucleus. (F) Mean fluorescence intensity of p-c-Jun expression in nuclei. (G, H) Release of TNF-*α* and IL-6 as detected by ELISA. Results are expressed as means ± S.E.M.*n* = 6. ^###^*P* < 0.001*vs.* control, ^∗^*P* < 0.05, ^∗∗^*P* < 0.01, and ^∗∗∗^*P* < 0.001*vs.* OGD/R. Supplementary Figure 3: the effect of tilianin on superoxide dismutase (SOD) and glutathione peroxidase (GSH-Px) activity after I/R injury in heart tissue and the inhibitory effect of KN93 on CaMKII kinase activity *in vitro*. The activities of SOD (A) and GSH-Px (B) in heart tissue were measured using the commercial assay kits (Nanjing Jiancheng Bioengineering Institute, Nanjing, China) according to the manufacturer's instructions (*n* = 4). (C) KN93 had the inhibitory effect on CaMKII activity *in vitro* (*n* = 5), and IC50 was 6.37 *μ*M. Results are expressed as the mean ± S.E.M.^###^*P* < 0.001 vs. control.
