# Supplementary material for: Tilianin Protects against Ischemia/Reperfusion-Induced Myocardial Injury through the Inhibition of the Ca2+/Calmodulin-Dependent Protein Kinase II-Dependent Apoptotic and Inflammatory Signaling Pathways
Source: Biomed Res Int. 2020 Oct 9;2020:5939715. doi: 10.1155/2020/5939715 (PMC7568786; doi:10.1155/2020/5939715)
Supplement: Supplementary Materials — Supplementary Figure 1: protein expression of CaMKII isoforms in H9c2 cardiomyocytes. Cell lysates were analyzed for CaMKIIα (A), CaMKIIβ (B), CaMKIIδ (C), and CaMKIIγ (D) expression by western blotting using the specific antibodies. GAPDH expression was used for comparison. Supplementary Figure 2: tilianin decreases JNK/c-Jun and NF-κB inflammatory signaling in OGD/R-injured H9c2 cardiomyocytes. (A) Representative images of p-p38, p-ERK1/2, p-JNK, p-c-Jun, and p-p65 expression (20x). (B-E) Mean_CircRingAvgIntenDiff values that describe the translocation of cytosolic p-p38 (B), p-ERK1/2 (C), p-JNK (D), and p-p65 (E) to the nucleus. (F) Mean fluorescence intensity of p-c-Jun expression in nuclei. (G, H) Release of TNF-α and IL-6 as detected by ELISA. Results are expressed as means ± S.E.M.n = 6. ###P < 0.001vs. control, ∗P < 0.05, ∗∗P < 0.01, and ∗∗∗P < 0.001vs. OGD/R. Supplementary Figure 3: the effect of tilianin on superoxide dismutase (SOD) and glutathione peroxidase (GSH-Px) activity after I/R injury in heart tissue and the inhibitory effect of KN93 on CaMKII kinase activity in vitro. The activities of SOD (A) and GSH-Px (B) in heart tissue were measured using the commercial assay kits (Nanjing Jiancheng Bioengineering Institute, Nanjing, China) according to the manufacturer's instructions (n = 4). (C) KN93 had the inhibitory effect on CaMKII activity in vitro (n = 5), and IC50 was 6.37 μM. Results are expressed as the mean ± S.E.M.###P < 0.001 vs. control. [file 5939715.f1.docx]

**Supplementary Files**

**Expression of Four Isoforms of CaMKII in H9c2 Cardiomyocytes**

The western blot assay showed the expression of CaMKIIδ in H9c2 cells was higher than the other three isoforms (Supplementary Fig. 1). Therefore, we explored the relationship between tilianin and CaMKIIδ in myocardial ischemia reperfusion injury in the following experiment.


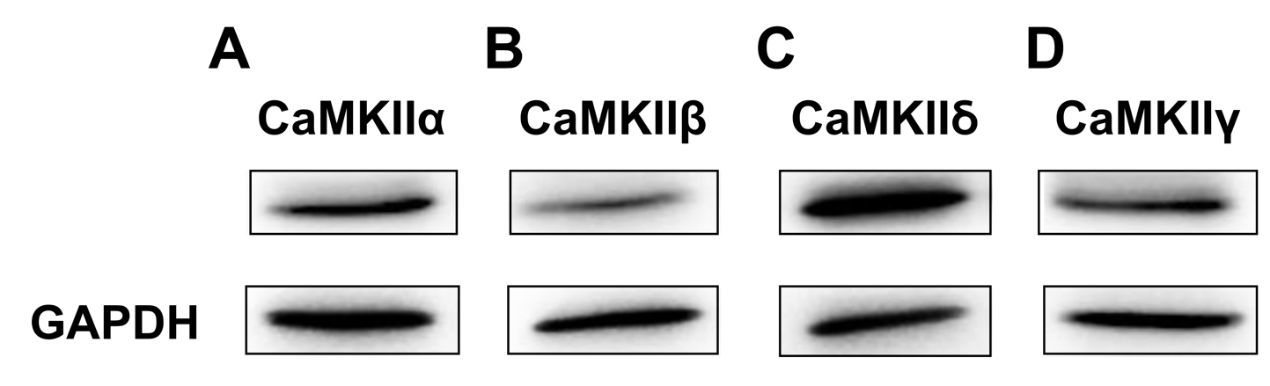


**Supplementary Figure 1. Protein expression of CaMKII isoforms in H9c2 cardiomyocytes.** Cell lysates were analyzed for CaMKIIα (A), CaMKIIβ (B), CaMKIIδ (C), and CaMKIIγ (D) expression by western blotting using the specific antibodies. GAPDH expression was used for comparison.

**Tilianin Decreases JNK/c-Jun and NF-κB Inflammatory Signaling in OGD/R-Injured H9c2 Cardiomyocytes**

OGD/R injury promoted the phosphorylation of MAPKs, demonstrated by a significant increase in Mean_CircRingAvgIntenDiff values indicating translocation of phosphorylated p38 (p-p38), phosphorylated extracellular signal-regulated kinase 1 and 2 (p-ERK1/2), and phosphorylated c-Jun N-terminal protein kinase (p-JNK) from the cytoplasm to the nucleus (Supplementary Fig. 2A-D, all *P*<0.001). The increased level of p-c-Jun, which acts as a p-JNK downstream substrate in the nucleus, was shown by an increased variation in mean fluorescent intensity (Supplementary Fig. 2A and E, *P*<0.001). The translocation of cytosolic p-JNK to the nucleus and the upregulation of p-c-Jun were both inhibited by tilianin at concentrations ranging from 0.8 μM to 100 μM in OGD/R-injured H9c2 cells (*P*<0.01-0.001), whereas there was no significant effect of tilianin on the nuclear translocation of p-p38 or p-ERK1/2.

The degree of NF-κB-mediated inflammatory response was quantified by assessment of translocation of cytosolic p65 to the nucleus and release of pro-inflammatory cytokines from H9c2 cells. Similarly, the translocation of phosphorylated p65 subunit (p-p65) from the cytoplasm to the nucleus and the TNF-α and IL-6 level in the culture supernatant were both increased in OGD/R-injured H9c2 cells (Supplementary Fig. 2A, F-H, *P*<0.05-0.001), but tilianin blocked p-p65 translocation and attenuated secretion of TNF-α and IL-6 (*P*<0.05-0.001). These results indicated that in H9c2 cells subjected to OGD/R-triggered toxicity, tilianin provided an anti-inflammatory effect via inhibition of the JNK/c-Jun pathway and NF-κB activation.





**Supplementary Figure 2. Tilianin decreases JNK/c-Jun and NF-κB inflammatory signaling in OGD/R-injured H9c2** **cardiomyocytes.** (A) Representative images of p-p38, p-ERK1/2, p-JNK, p-c-Jun, and p-p65 expression (20×). (B-E) Mean_CircRingAvgIntenDiff values that describe the translocation of cytosolic p-p38 (B), p-ERK1/2 (C), p-JNK (D), and p-p65 (E) to the nucleus. (F) Mean fluorescence intensity of p-c-Jun expression in nuclei. (G, H) Release of TNF-α and IL-6 as detected by ELISA. Results are expressed as means ± S.E.M. *n*=6. ^###^*P*<0.001 *vs.* control, **P*<0.05, ***P*<0.01, ****P*<0.001 *vs.* OGD/R.

**Effects of Tilianin on Superoxide Dismutase (SOD) and Glutathione Peroxidase (GSH-Px) Activity after I/R Injury in Heart Tissue and Effect of KN93 on CaMKII Kinase Activity *In Vitro***

Protection of antioxidation capacity, indicated by SOD and GSH-Px activity, due to treatment with tilianin was not improved when isolated hearts were subjected to I/R injury (Supplementary Fig. 3A-B). The CaMKII inhibitor KN93 demonstrated the inhibitory effect on CaMKII activity with an IC50 6.37 μM *in vitro* (Supplementary Fig. 3C).





**Supplementary Figure 3. The effect of tilianin on superoxide dismutase (SOD) and glutathione peroxidase (GSH-Px) activity after I/R injury in heart tissue and the inhibitory effect of KN93 on CaMKII kinase activity *in vitro*.** The activities of SOD (A) and GSH-Px (B) in heart tissue were measured using the commercial assay kits (Nanjing Jiancheng Bioengineering Institute, Nanjing, China) according to the manufacturer’s instructions (*n*=4). (C) KN93 had the inhibitory effect on CaMKII activity *in vitro* (*n*=5) and IC50 was 6.37 μM. Results are expressed as mean ± S.E.M. ^###^*P*<0.001 vs. control.
